# Proof of Principle for Direct Reconstruction of Qualitative Depth Information from Turbid Media by a Single Hyper Spectral Image

**DOI:** 10.3390/s21082860

**Published:** 2021-04-19

**Authors:** Martin Hohmann, Damaris Hecht, Benjamin Lengenfelder, Moritz Späth, Florian Klämpfl, Michael Schmidt

**Affiliations:** 1 Institute of Photonic Technologies (LPT), Friedrich-Alexander-Universität Erlangen-Nürnberg (FAU), Konrad-Zuse-Strasse 3/5, 91052 Erlangen, Germany; Damaris.Hecht@FAU.de (D.H.); Benjamin.Lengenfelder@lpt.uni-erlangen.de (B.L.); Moritz.Spaeth@lpt.uni-erlangen.de (M.S.); Florian.Klaempfl@lpt.uni-erlangen.de (F.K.); Michael.Schmidt@lpt.uni-erlangen.de (M.S.); 2 Erlangen Graduate School in Advanced Optical Technologies (SAOT), Paul-Gordon-Strasse 6, 91052 Erlangen, Germany

**Keywords:** hyperspectral, hyperspectral depth reconstruction, HSDR, hyperspectral imaging

## Abstract

In medical applications, hyper-spectral imaging is becoming more and more common. It has been shown to be more effective for classification and segmentation than normal RGB imaging because narrower wavelength bands are used, providing a higher contrast. However, until now, the fact that hyper-spectral images also contain information about the three-dimensional structure of turbid media has been neglected. In this study, it is shown that it is possible to derive information about the depth of inclusions in turbid phantoms from a single hyper-spectral image. Here, the depth information is encoded by a combination of scattering and absorption within the phantom. Although scatter-dominated regions increase the backscattering for deep vessels, absorption has the opposite effect. With this argumentation, it makes sense to assume that, under certain conditions, a wavelength is not influenced by the depth of the inclusion and acts as an iso-point. This iso-point could be used to easily derive information about the depth of an inclusion. In this study, it is shown that the iso-point exists in some cases. Moreover, it is shown that the iso-point can be used to obtain precise depth information.

## 1. Introduction

The use of optics in medicine has become increasingly important in recent decades. This can be seen, among other things, in the increasing number of lasers used in hospitals [1]. The same trend can be observed in medical imaging. It has its application both in diagnostics and therapeutics [2,3,4,5]. For medical diagnostics, imaging is probably the most important technique. Ideally, medical imaging provides the physician a two- or three-dimensional representation of disease-related parameters. The most important approach for imaging modalities is optical imaging.

The two gold standard methods for optical imaging of tissue are optical coherence tomography (OCT) and photo acoustic tomography (PAT). In PAT, turbid media is illuminated with short pulsed laser light, which is absorbed and converted into heat. The heating causes the tissue to expand locally. This produces pressure waves that can be detected with ultrasound detectors at the surface. There are also approaches for purely optical detection [6,7]. The measurement time for this can be quite long: an image of the female breast takes about 3 min with an additional reconstruction time of 25 min [8]. However, in more recent approaches, imaging speeds of up to 10 Hz are possible [9,10]. OCT, as the second gold standard for tomographic imaging of turbid media, allows imaging with a resolution of up to 1 μm [11] by measuring the back reflection of a low coherence light source with an interferometer. Also, a high imaging speed of up to 4.5 Gvoxels/s is possible [12,13,14,15]. However, the resolution drops to 10 μm for ultrafast OCTs. While both PAT and OCT show excellent results, the amount of information that can be collected is limited since they use either absorption or scattering as a contrast source.

In addition to OCT and PAT, diffuse reflectance techniques can also be used to obtain 3D information about the turbid media under investigation. It has been shown for a two-layer turbid medium that it is possible to measure the scattering and absorption properties of both layers by illuminating them with two different wavelengths. However, this approach has the disadvantage of being dependent on a particular model [16]. This model assumes that each wavelength propagates in both layers. However, due to the different penetration depth of the two wavelengths, the relative contribution of the two layers to the reflection varies.

Using these relative contributions, depth information can be calculated. In addition to the reconstruction, it can be seen from the study of Saager et al. [16] that the varying penetration depths of the different wavelengths leads to a different information composition of the diffuse reflection on the surface. When the wavelength bands considered are narrow and spectrally close enough, the mixing between the depth information for two adjacent wavelength bands might be linear. Therefore, it is deemed possible with hyper-spectral imaging (HSI) to reconstruct the depth information.

So far, HSI has shown that reconstruction of the subsurface, objects, and vegetation in the ocean [17] can be achieved by using wavelengths with different penetration depths for purely absorbing media. In a previous publication [18], it was shown by our group that for two inclusions with different depths, the spectra change for different inclusion depths. The principle effect can also be seen in the study by Zherebtsov et al. [19]. They used extensive Monte Carlo simulations and a neural network to measure spatially resolved epidermal thickness, melanin content, and blood volume fraction. In their Figure 6, it can be seen that the spectra change significantly for different depth levels. In the spectral range from 550 nm to 600 nm (absorbing range), the reflectance is higher for lower inclusions.

In this study, the qualitative results of our old study [18] are extended since it is shown that under certain conditions it is possible to derive clear depth information from a single hyper-spectral image. Thereby, as known from the literature, the depth of an inclusion affects the reflectance spectra. In general for turbid media, an absorber will appear dark at the surface, while a region of stronger scattering inside the turbid medium will appear bright at the surface. In between, there may be a point where the two effects cancel each other out. With this argumentation it makes sense to assume that under certain conditions a wavelength is not influenced by the depth of the inclusion and acts as an iso-point. This iso-point could be used to easily derive information about the depth of an inclusion. In this study, it is shown that the iso-point exists. Moreover, it is shown that the iso-point can be used to obtain precise depth information.

## 2. Methods

### 2.1. Set-Up and Phantom

The phantom used for imaging is made from Polyurethane (WC-781, BJB Entprise Co., Tustin, CA, USA) with titanium oxide powder (Sigma-Aldrich GmbH, Darmstadt, Germany) and red ink (Pelikan GmbH, Hannover, Germany) as scatterer and absorber, respectively. Thereby, the concentrations of the scatterer and absorber are chosen to result in lower absorption and scattering coefficients than tissue. This is done to simplify the manufacturing process of the inclusions. As the scattering and absorption are lower, all effects happen at a larger scale. Thus, the inclusion can be larger as well.

**Figure 1:**
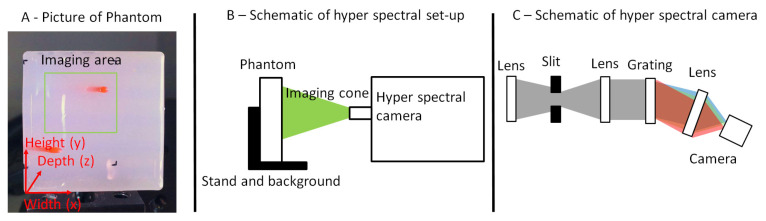
(**A**): Sample phantom with inclusions filled with a red ink water solution. The green rectangle shows the part of the phantom for which the data analysis is done. Only the upper inclusion was used for the analysis as the lower one would be prone to boundary effects. The inclusion has a diameter of two millimeters and it is drilled from the top to the bottom surface. On the right part, it crosses the top surface (closer to camera) while in the left part, it crosses the bottom surface (further away from the camera). (**B**): Set-up. The phantom is illuminated from the side and imaged with the hyper-spectral camera (adapted from [18]). (**C**): Schematic of the set-up of the hyper-spectral camera.

Figure 1 shows the phantom used in this study. The inclusions are filled with a mixture of water and ink with an ink concentration of 0%, 1%, and 10%. For the first analysis, the 10% ink concentration was used. For imaging, only the left part of the phantom was used due to the fact that this is the field-of-view of the camera. For the analysis, only the top drilling was chosen to minimize boundary effects from the phantom. The inclusion has a diameter of two millimeters and it was drilled from the top to the bottom surface. In the right part, it crosses the top surface (closer to camera), whereas in the left part, it crosses the bottom surface (further away from the camera). Additionally, the depth of the inclusion was measured with a combination of calliper and OCT (Telesto II, Thorlabs, Newton, NJ, USA). Hence, the precision of the drilling has no significant relevance as it is characterized afterwards. This result was used to generate a depth map of the whole phantom to validate the method later on.

This phantom was imaged with a self-built hyper-spectral camera shown in our old study [18]. It is solely built for phantom imaging. The camera system is not designed for later in vivo usage as our group puts emphasis on endoscopic applications [20,21,22,23]. The camera system consists of a lens imaging the phantom on a slit, which is collimated and dispersed with a grating. After the grating, the light was imaged on a CMOS camera. The resulting voxel resolution is x×y×λ=95×500×950voxel. The pixel size in x direction corresponds to 260 μm and in y direction to 43 μm. The spectral range is 407 nm–670 nm. This leads to $0.3\;\frac{nm}{pixel}$. However, the effective spectral resolution is only around 10 nm. Thus, spectral averaging can be done without loss of information. It should be noted that this resolution is sufficient, as the used absorber has a peak with a spectral width of around 100 nm.

Current imaging errors in the final hyper-spectral image are partly caused by random noise from the detector system and partly by the keystone and smile. Compared to our old study [18], the imaging noise is much smaller due to another light source with a much higher output power. For the hyper-spectral camera used, keystone is more dominant than smile. Moreover, the effect of both of them is minimized by performing the analysis in a small part of the image. Furthermore, their effect is nearly completely compensated by doing the main spectral analysis perpendicular to the direction in which the smile and the keystone alter the spectrum.

The used light source is a passively cooled LED based light source (Plant Light Science GmbH & Co. KG, Veitsbronn, Germany) in the spectral range from 400 nm–670 nm. Thereby, the spectrum can be arbitrarily adjusted. It is adjusted to be as flat as possible to simplify the hyper-spectral measurement. The spectrum is shown in attachment A. The light source is place to illuminate the sample from the top under an angle of around 40°.

### 2.2. Depth Effect on Reflectance Spectrum

**Figure A1:**
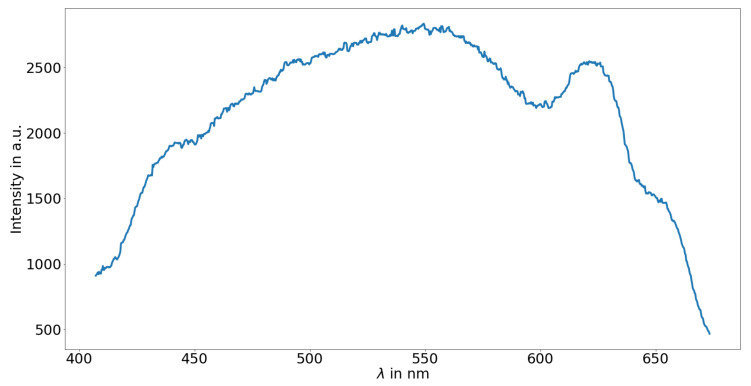
Reflectance spectrum from barium sulfate measured by the hyper-spectral camera before normalization. The drop of intensity in the blue and the red region is caused by the spectral efficiency of the grating and the camera.

For the data analysis of the depth effect, every spectrum is divided by a reference spectrum measured from a barium sulfate plate as spectral normalization (Figure A1). Before this normalization step, both spectra are filtered by a median filter with a size of 5×5 pixels for noise reduction. In general, the noise is rather small due to the strong light source. Thus, an elaborated noise filtering is not required. However, the strong light source illuminates the complete room. Therefore in combination with the spatial scanning approach, unwanted movements of the experimentalist might influence the signal in x-direction. To compensate this, a median filter was chosen.

### 2.3. Reconstruction of Depth Information

In general, it is expected that for lower wavelengths the absorption is dominant as a red absorber is used. For longer wavelengths, the effect of the scattering is expected to be more pronounced as no matching absorber is present in this spectral range. Between both of these spectral ranges, there might be an iso-point on which the depth of the inclusion does not influence the spectrum on the surface. In this study, the iso-point is at 575 nm. Thus, by Equation (1), the depth information can be calculated:

(1) $$di=1-\frac{I_{550\;\mathrm{nm}}}{I_{575\;\mathrm{nm}}}$$

where $I_{575\;\mathrm{nm}}$ is the reflectance at 575 nm, $I_{550\;\mathrm{nm}}$ is the reflectance at 550 nm and di is the depth information. Equation (1) is defined to be zero without inclusion and rises to more positive values with increasing depth. It should be noted that other spectral ranges could also be selected instead of 550 nm. For noise reduction, twenty neighboring spectra are averaged. This should not influence the spectral information due to the fact that the spectral range of twenty wavelengths is around 6 nm, which is less than the spectral resolution of 10 nm. For the generation of the image wide depth information from Equation (1), the calculation is done for the whole image.

## 3. Results

The results section is divided into two parts. In the first part, the spectra are characterized to discuss the presence and existence of the iso-point. In the second part, the reconstruction is performed with the help of the iso-point and evaluated.

### 3.1. Depth Effect on Reflectance Spectrum

**Figure 2:**
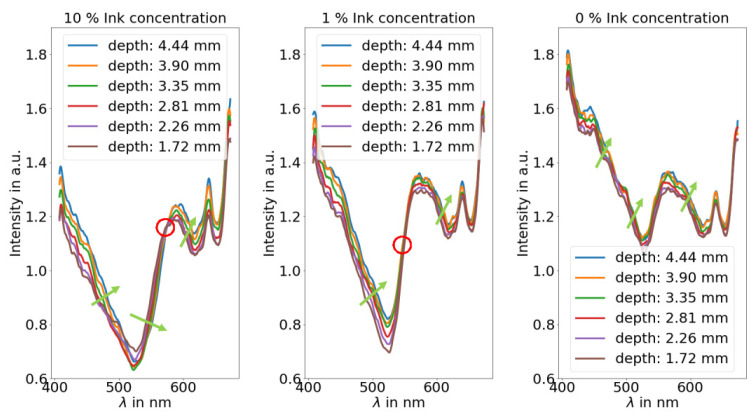
Spectra for different x-directions and, therefore, different inclusion depths as a function of the ink concentration inside the inclusions. The lower the x-position, the deeper the inclusion is. The green arrow symbolizes the spectra for deeper inclusions and the red circle symbolizes the position of the iso-point (10% ink) or the position there is nearly not depth effect (1% ink). With water inside the inclusion, there is no iso-point.

Figure 2 shows the spectra for different ink concentrations and different x-directions and, therefore, different depths. The lower the x-position, the deeper the inclusion is.

It can be seen that there is an iso-point if the ink concentration is high enough. For 10% ink concentration, there is an iso-point while for 1% it is at the boundary (the spectra touch in one point) and for 0% ink concentration there is no iso-point. Hence, an absorption dominated regime and a scattering dominated regime are required. It can be concluded that strong absorption in the inclusion might be a necessary condition for the iso-point. For the spectral behavior with 10% ink, it can be seen that the reflection increases for deeper inclusions with longer wavelengths than 575 nm. For below 575 nm, the opposite effect is present: The reflection increases for deeper inclusions. While the first effect is independent of the absorber in the inclusion, the latter depends on the absorber concentration and, therefore, the ink concentration in the inclusion. Thus, there is an absorbing regime and a scattering regime.

In this study, a red absorber was used; therefore, the absorption coefficient of the used ink is high in the blueish and greenish region while it drops significantly in the reddish and near-infra-red region. Hence, the blueish and greenish regions are seen as absorbing regions, whereas the reddish and near-infra-red regions are seen as scattering regions. In the absorbing region, the appearance of the inclusion becomes darker as it is closer to the surface. This happens due to the fact that more light is absorbed when the inclusion is closer to the surface. In the scattering regime, the opposite happens. The inclusion appears brighter when it is closer to the surface. This effect can occur by an increase of the scattering coefficient in the inclusion, by surface scattering at the boundaries or simply by Fresnel reflection due to an index mismatch at the boundaries. If the absorption coefficient gradually increases (shorter wavelength), the reduction of back reflection due to absorbance increases. At a certain point, it will overshadow the increase of reflection by the scattering coefficient. If the increase for the absorption coefficient continues, there will be a point for which both effects compensate. The spectral position of the iso-point is expected to depend on the absorption and scattering coefficient.

The ink dependence leads to two conclusions: First, the lower reflection for higher inclusions is caused by the absorber in the inclusion. Second, as the difference is rather small, this effect does not prevent usage in in vivo settings as the hemoglobin concentration in a blood vessel only varies by less than a factor of two between different humans. Moreover, with water inside the inclusion, there is no iso-point. Therefore, the absorption in the inclusion is a necessary condition for the iso-point.

Furthermore, it should be noted that there is currently no assumption about the scattering. The only requirement is for the absorption. Within the measured spectral range in one spectral part, the absorption has to be dominant and in another neighboring spectral range, the scattering has to be dominant. Nevertheless, this situation is present for tissue with hemoglobin as absorber. The results of Zherebtsov et al. [19] also suggest that the iso-point might be present. Hence, it is likely that other chromophores will work. More importantly, this also means that for realistic optical properties the presented method might work. Moreover inside the inclusions, there has to be an absorber most likely the same as in the substrate. However, this should not be a limitation for in-vivo imaging as this situation occurs with hemoglobin in tissue.

### 3.2. Reconstruction of Depth Information

**Figure 3:**
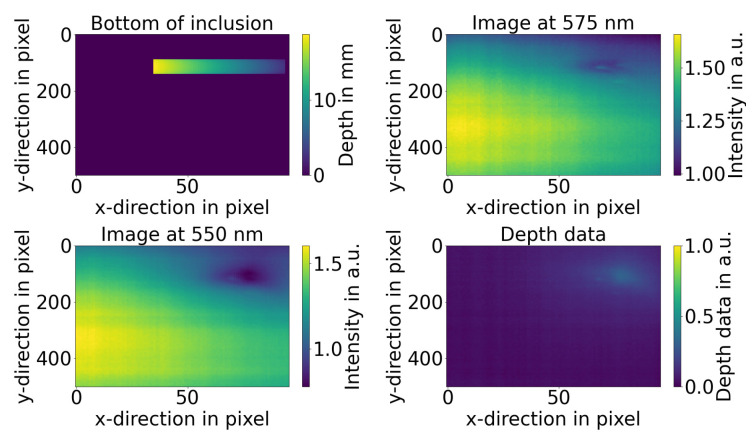
Comparison of the inclusion measured by OCT and caliper (**top left**) with the hyper-spectral signal at 550 nm (**bottom left**) and the hyper-spectral signal at the iso-point at 575 nm (**top right**) with the depth map (**bottom right**).

Figure 3 shows a comparison of the inclusion (top left) with the signal at 550 nm (bottom left) and the averaged signal at 575 nm (top right) with the depth map (bottom right). It can be seen that the spectrum at 575 nm is barely influenced by the inclusion depth at all while the spectra at 550 nm shows a strong influence. These data show that the iso-point is present.

**Figure 4:**
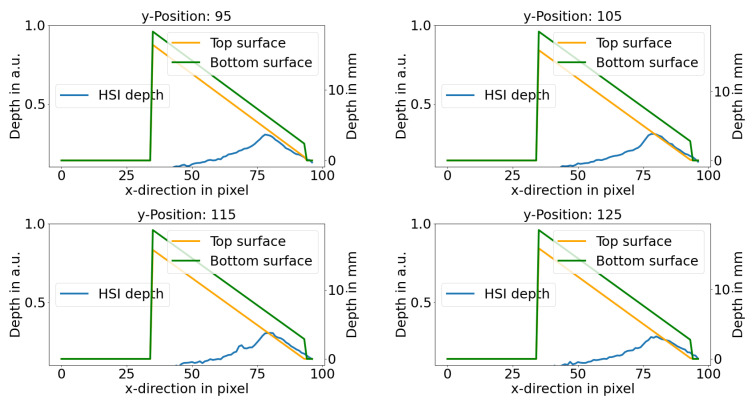
HSI depth parameter along the inclusion for different positions perpendicular to the inclusion and the lower and upper depth of the inclusion. The left y-axis shows the depth parameter and the right y-axis the results from the real depth. In all cases, the depth parameter acts linear (marked by red ellipses) with the actual depth up to a maximal depth depending on the optical properties. The green and orange graphs represent the real depth, measured by an OCT and a caliper.

For a more detailed inspection, Figure 4 shows the HSI depth parameter for different positions along the inclusion. It can be seen that there is a linear behavior between the depth parameter and the real depth. The linearity is present until a inclusion depth of up to 6.5 mm or 3.5 mm for the lower or upper surface of the inclusion, respectively. This depth is also the spectral range in which the iso-point starts to disappear. For larger inclusion depths as a result, the linearity of the depth parameter fails and shows an behavior related to an exponential decrease. The results from both regimes are analyzed later in this study in more detail. Hence, there is a linear and an exponential depth regime that behaves similarly to the behavior of a quadratic function $y=x^{2}$. Nevertheless, it is not clear how the results for the exponential regime can be interpreted as the iso-point is not present. However, it can be concluded that the presented iso-point method does not work in the whole depth range.

**Figure 5:**
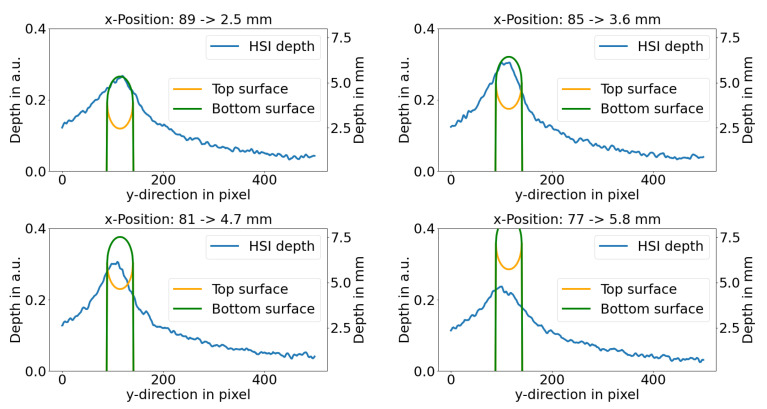
HSI depth parameter perpendicular to the inclusion for different positions along the inclusion and the lower and upper depth of the inclusion. The left y-axis shows the depth parameter and the right y-axis the results from the real depth. The green and orange graphs represent the real depth, measured by an OCT and a caliper.

Figure 5 shows the depth parameter perpendicular to the inclusion for different positions in x-direction and, therefore, different inclusion depths. Moreover, it can be seen that the inclusion size appears much larger than the actual size of the inclusion due to the blurring caused by the scattering. It can be concluded that the depth reconstruction method presented in this study may be limited to a rather sparse amount of inclusion. Only in this case, a precise depth can be reconstructed.

**Figure 6:**
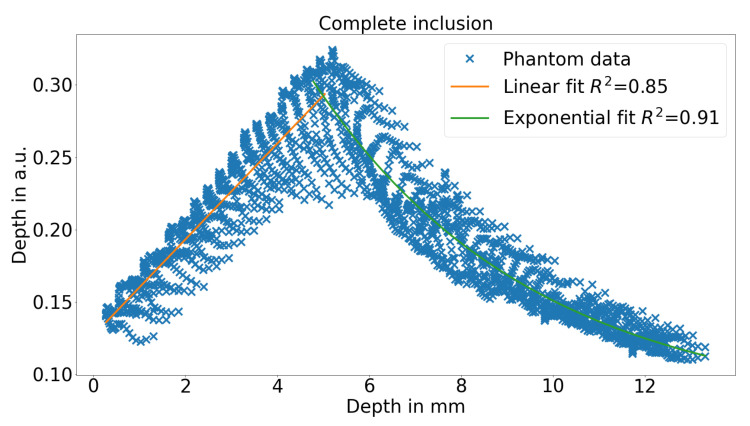
HSI depth parameter in the linear regime and the exponential regime over the real depth of the bottom channel with the according fits.

**Figure 7:**
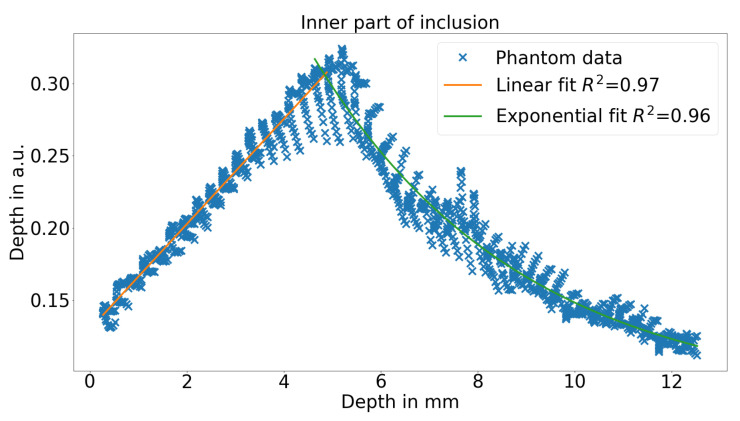
HSI depth parameter in the linear regime and the exponential regime over the real depth of the bottom channel with the according fits for the central part of the inclusion.

Figure 6 shows the relationship for the depth parameter and the real depth of the inclusions. On the left side, the linear regime is shown and on the right side, the exponential regime is shown. In the linear and exponential regime 85% and 91% of the variance can be explained by the fit. These fairly low values are mainly caused by the fact the the boundaries of the inclusion cannot be assessed by the presented method. However, if only the inner 60% of the inclusion is used, the fit will describe more than 95% of the variance as seen in Figure 7.

Furthermore, it can be seen that especially close to the surface, the agreement is comparably high and it degrades when the channel is deeper.

## 4. Conclusions

In summary, hyper-spectral imaging can be used to reconstruct depth information of inclusions in a turbid medium. Furthermore, for a certain depth range of an inclusion, an iso-point is present. In general, there is an absorption effect and a scattering effect that have opposite effects on the measured spectra. The absorber in the inclusion absorbs the light and, therefore, reduces the back reflection especially for lower depths of the inclusions. In the non-absorbing spectral region, the back reflection is stronger for deeper inclusions. Thus, the two effects are opposite. In addition, there is an iso-point in between both spectral ranges where both depth-dependent effects cancel each other out. Furthermore there is a maximum depth up to which the analysis scheme works: in this case about 4–6 mm. However, the possible depth depends on the optical properties. Since a lower scattering and absorber concentration is used in this study, it is realistic to assume that with optical properties similar to those of tissue, the depth could be a factor of three smaller.

The main drawbacks are the surjective mapping behavior between the depth parameter and the actual inclusion depth, similar to the behavior of a quadratic function $y=x^{2}$, which is caused by the fact the iso-point disappears for high depths. Furthermore, the proposed method will not work in the full range of optical properties. One possible solution to this problem is to change the optical properties of the tissue. For example, scattering within blood vessels can be increased using microbubbles. Normally there is no strong scattering inside a blood vessel. However, this can be can be compensated for by the use of microbubbles, which greatly increase the scattering [24,25]. Alternatively, the scattering of the tissue can also be reduced by optical clearing or the absorption properties can be increased by staining.

## Data Availability

The data that supports the findings of the study are provided within the article. The raw data is available upon request to the corresponding author.

## References

[1] Späth M., Klämpfl F., Stelzle F., Hohmann M., Lengenfelder B., Schmidt M. (2019). A quantitative evaluation of the use of medical lasers in German hospitals. J. Biophotonics.

[2] Vakoc B., Fukumura D., Jain R., Bouma B. (2012). Cancer imaging by optical coherence tomography: Preclinical progress and clinical potential. Nat. Rev. Cancer.

[3] Doi K. (2007). Computer-aided diagnosis in medical imaging: Historical review, current status and future potential. Comput. Med. Imaging Graph..

[4] Lu G., Fei B. (2014). Medical hyperspectral imaging: A review. J. Biomed. Opt..

[5] Prince J., Links J. (2006). Medical Imaging Signals and Systems.

[6] Lengenfelder B., Mehari F., Hohmann M., Heinlein M., Chelales E., Waldner M., Klämpfl F., Zalevsky Z., Schmidt M. (2019). Remote photoacoustic sensing using speckle-analysis. Sci. Rep..

[7] Shabairou N., Lengenfelder B., Hohmann M., Klämpfl F., Schmidt M., Zalevsky Z. (2020). All-optical, an ultra-thin endoscopic photoacoustic sensor using multi-mode fiber. Sci. Rep..

[8] Kruger R., Kuzmiak C., Lam R., Reinecke D., Del Rio S., Steed D. (2013). Dedicated 3D photoacoustic breast imaging. Med. Phys..

[9] Buehler A., Deán-Ben X., Claussen J., Ntziachristos V., Razansky D. (2012). Three-dimensional optoacoustic tomography at video rate. Opt. Express.

[10] Dima A., Ntziachristos V. (2012). Non-invasive carotid imaging using optoacoustic tomography. Opt. Express.

[11] Bizheva K., Haines L., Mason E., MacLellan B., Tan B., Hileeto D., Sorbara L. (2016). In Vivo Imaging and Morphometry of the Human Pre-Descemet’s Layer and Endothelium With Ultrahigh-Resolution Optical Coherence TomographyIn Vivo UHR-OCT of Posterior Corneal Layers. Investig. Ophthalmol. Vis. Sci..

[12] Wieser W., Biedermann B., Klein T., Eigenwillig C., Huber R. (2010). Multi-megahertz OCT: High quality 3D imaging at 20 million A-scans and 4.5 GVoxels per second. Opt. Express.

[13] Kolb J., Klein T., Wieser W., Draxinger W., Huber R. (2015). Full volumetric video rate OCT of the posterior eye with up to 195.2 volumes/s. SPIE BiOS.

[14] Zhi Z., Qin W., Wang J., Wei W., Wang R. (2015). 4D optical coherence tomography-based micro-angiography achieved by 1.6-MHz FDML swept source. Opt. Lett..

[15] Wieser W., Draxinger W., Klein T., Karpf S., Pfeiffer T., Huber R. (2014). A 4-D OCT Engine with 1 GVoxel/s. Opt. Photon. News.

[16] Saager R., Truong A., Cuccia D., Durkin A. (2011). Method for depth-resolved quantitation of optical properties in layered media using spatially modulated quantitative spectroscopy. J. Biomed. Opt..

[17] Bostater C., Jones J., Frystacky H., Kovacs M., Jozsa O. (2010). Image analysis for water surface and subsurface feature detection in shallow waters. Remote Sensing.

[18] Hohmann M., Hecht D., Lengenfelder B., Klämpfl F., Schmidt M. (2019). Direct reconstruction of qualitative depth information from turbid media by a single hyper spectral image. Optical Diagnostics and Sensing XIX: Toward Point-of-Care Diagnostics.

[19] Zherebtsov E., Dremin V., Popov A., Doronin A., Kurakina D., Kirillin M., Meglinski I., Bykov A. (2019). Hyperspectral imaging of human skin aided by artificial neural networks. Biomed. Opt. Express.

[20] Hohmann M., Kanawade R., Klämpfl F., Douplik A., Mudter J., Neurath M., Albrecht H. (2017). In-vivo multispectral video endoscopy towards in-vivo hyperspectral video endoscopy. J. Biophotonics.

[21] Hohmann M., Albrecht H., Mudter J., Nagulin K., Klämpfl F., Schmidt M. (2019). Spectral Spatial Variation. Sci. Rep..

[22] Ganzleben I., Hohmann M., Grünberg A., Menezes J.G., Vieth M., Liebing E., Günther C., Thonn V., Beß D., Becker C. (2020). Topical application of Chlorin e6-PVP (Ce6-PVP) for improved endoscopic detection of neoplastic lesions in a murine colitis-associated cancer model. Sci. Rep..

[23] Hohmann M., Albrecht H., Lengenfelder B., Klämpfl F., Schmidt M. (2020). Factors influencing the accuracy for tissue classification in multi spectral in-vivo endoscopy for the upper gastro-internal tract. Sci. Rep..

[24] Assadi H., Karshafian R., Douplik A. (2014). Optical scattering properties of intralipid phantom in presence of encapsulated microbubbles. Int. J. Photoenergy.

[25] Assadi H., Demidov V., Karshafian R., Douplik A., Vitkin I. (2016). Microvascular contrast enhancement in optical coherence tomography using microbubbles. J. Biomed. Opt..

